# Realizing the Improvement of Green Total Factor Productivity of the Marine Economy—New Evidence from China’s Coastal Areas

**DOI:** 10.3390/ijerph19148619

**Published:** 2022-07-15

**Authors:** Wenhan Ren, Yu Chen

**Affiliations:** Business School, Qingdao University, Qingdao 266061, China; chnyu0211@163.com

**Keywords:** marine economy, green total factor productivity, influence mechanism, direct effect, threshold effect

## Abstract

Paying attention to the mechanisms of the *GTFP* of the marine economy and designing a scientific and reasonable optimization path are the keys to achieving a “win-win” balance between environmental protection and high-quality marine development. Therefore, this paper considers the rigid constraints of resources and negative environmental effects to construct a multi-factor evaluation model of the *GTFP* of the marine economy including capital, labor, and resources to expand the evaluation method system for the sustainable development of the marine economy. On this basis, this paper determines the influencing factors of the *GTFP* of China’s marine economy, qualitatively analyzes the mechanism of each influencing factor on the *GTFP* of the marine economy, uses multi-dimensional data of coastal areas, quantitatively analyzes the direct and indirect effects of the factors that influence the *GTFP*, and proposes practical optimization paths and safeguarding measures, which provide a decision-making reference for the implementation of China’s marine development strategy. The results showed that the *GTFP* of China’s marine economy was in a state of improvement and increased from 0.9878 in 2006 to 1.2789 in 2018. The direct effects of environmental regulations have a negative and significant impact on *GTFP*, whereas economic development, human capital, and technological innovations have a positive and significant impact on *GTFP*. In addition, environmental regulations have an “inclined N” double-threshold effect on *GTFP*. The impact of environmental regulations on the *GTFP* of the marine economy depends on the intensity of the environmental regulations, as different intensities of environmental regulations have different dominant levels of the “innovation compensation effect” and “offset effect” that affect the *GTFP* of the marine economy.

## 1. Introduction

As China’s economy has gradually entered a new stage of improving quality and efficiency, high-quality development has become the theme of China’s present and future economic construction [1]. As a strategic location for high-quality development, the ocean plays a pivotal role in cultivating new growth drivers, expanding emerging industries, and leading new economic development [2]. The marine economy is a variety of related economic activities through the development and utilization of marine resources and marine spaces with the goal of obtaining economic benefits. Therefore, accelerating the realization of the high-quality development of the marine economy is not only in line with the requirements of the new era for the implementation of China’s marine power strategy but also improves China’s economic vitality and promotes China’s economic transformation and development [3].

High-quality development is a new type of development that adheres to quality first and benefits priority [4,5]. For a long time, China’s marine economy has been relying on the input of capital, labor, and other factors to achieve wealth creation and economic growth through the expansion of scale [6]. However, this mode of growth is ultimately insufficient and unsustainable [7]. With the rise in labor costs and the strengthening of resource and environmental constraints, both the macro-economy and micro-individuals urgently need to find a new driving force for development to solve the problem of the insufficient momentum of growth in the traditional economy. The high-quality development of the marine economy emphasizes the dynamic balance of the “economy–society–ecological environment” system [8]. To achieve this, continuously improving the total factor productivity (TFP) of the marine economy is the key or even the only way [9,10]. From the report of the 19th National Congress of the Communist Party of China, it can also be seen that the core approach to China’s economy from high-speed growth to high-quality development is to change from the unsustainable old driving force relying on the expansion of factor input to the sustainable new driving force mainly relying on TFP. Therefore, the high-quality development of the marine economy urgently needs to transform from extensional growth to connotative development, that is, to transform from relying mainly on the increase of production factor input to the improvement of TFP.

TFP is also called multifactor productivity [6]. Because its size is difficult to measure directly, there is no clear dimensional unit and it often gives the impression of being complex and mysterious. In order to better understand TFP, we might as well start with the concept of productivity. Productivity refers to the output that can be obtained by a given set of factor inputs and it measures the production efficiency of economic units (large for countries, small for factories and workshops). When there is only one input and output, the measure of productivity is very simple and can be expressed as the input–output ratio in mathematical form. However, actual economic production is not only a kind of input. In order to measure the production efficiency more objectively, it is necessary to measure the output that can be obtained by all the observable combinations of factor inputs so that there is TFP [11]. TFP can fully reflect the overall conversion efficiency of the system’s inputs into outputs and objectively reflect the comprehensive macroeconomic benefits of an economic system. As a tool to evaluate the source of economic growth, TFP has always attracted attention from all sectors of society [12,13,14,15,16]. Economies, regions, and enterprises are thinking in the long term about how to increase productivity and production quality without increasing production costs [17]. However, the measurement of TFP in the early literature was only based on the input of traditional factors such as capital and labor, without considering the depletion of resources and the deterioration of the ecological environment [18]. The huge resource and environmental costs during the process of economic development were reflected in the gross domestic product (GDP) in the form of economic growth; such research results cannot faithfully reflect the real situation of economic development [19].

With the increasingly serious constraints of ecological and environmental problems in sustainable development, some scholars have begun to incorporate environmental pollution factors into the traditional analysis framework of TFP to measure green total factor productivity (*GTFP*) [20]. As a new form of traditional TFP from the perspective of ecological civilization, *GTFP* has been widely used in the consideration of the coordinated development of the economy and environment [21,22]. Subsequently, different organizations defined *GTFP* separately [23,24]. The different definitions have common connotations and their core ideas are low investment, low pollution, and high returns. The green total factor productivity defined in this paper is based on the traditional total factor productivity, which takes resource consumption as the input factor and environmental pollution emissions as the undesired output for the productivity accounting framework system. Based on the connotations of *GTFP*, existing studies have begun to focus on the evaluation model, temporal and spatial evolution law, and agglomeration status of *GTFP* [2,25,26,27,28,29,30]. However, existing research mainly focuses on land areas such as the logistics industry, agriculture, and the tourism industry [31,32,33,34]. There is less research in marine areas, especially research that is devoted to the marine economy as a whole. In fact, the marine economy is a resource-based economy in the traditional sense. The development of marine resources has formed traditional and emerging marine industries. Marine resources and the environment are not only the material basis of the marine economy but are also the restrictive factors of coastal economic and social development [35,36]. In terms of input, most of the existing research considers the input of traditional factors such as marine capital and labor, ignoring the rigid constraints of marine resources. In terms of output, most of the existing studies are measured by the desirable output of gross ocean product (GOP). Although some scholars have considered the impact of undesired output on marine economic efficiency, most of them use a single pollutant emission directly as an undesired output to be substituted into the model, which is not scientific and comprehensive. In the measurement method, the more traditional DEA and SFA methods are mainly used. Although the methods are relatively mature, they lack consideration of non-radial, non-angular, and mixed-radial problems, resulting in a certain deviation between the efficiency measurement results and the actual situation [37,38]. In addition, most of the literature on *GTFP* in the marine field calculates the efficiency value based on the perspective of factor input and output [25,26,27,28,29,32,34]. However, the *GTFP* of the marine economy is not only a problem between input and output but is also affected by external factors and will change with these factors. Therefore, in order to achieve a “win-win” balance between environmental protection and marine economic development, besides accurately measuring and grasping the current situation and regional differences of the *GTFP* of the marine economy, it is also necessary to find out what are the external factors that affect *GTFP* and what are the mechanisms of each factor that influence the *GTFP* of the marine economy. Only by solving these problems can we find the most scientific and reasonable optimization path to improve the *GTFP* of the marine economy and the current situation of China’s marine environment.

Therefore, this paper combines the characteristics of the marine economy and the requirements of high-quality development and considers the rigid constraints of resources and negative environmental effects to construct a multi-factor evaluation model of the *GTFP* of the marine economy including capital, labor, and resources. On this basis, this paper determines the influencing factors of the *GTFP* of China’s marine economy, qualitatively analyzes the mechanisms of each influencing factor on the *GTFP* of the marine economy, uses multi-dimensional data of coastal areas, and quantitatively analyzes the direct effect of the influencing factors on *GTFP*. In addition, this paper further discusses whether there is a “Porter Hypothesis” in the marine field and tries to unravel the mystery of the changes in the *GTFP* of the marine economy. The research objectives of this paper mainly include three aspects: (1) to improve the research level of the quantification and refinement of the *GTFP* of the marine economy and expand the evaluation method system for the sustainable development of the marine economy; (2) to answer the temporal and spatial differences of the *GTFP* of China’s marine economy, understand the practical problems existing in the process of China’s marine economic growth, and clarify the future development direction of China’s marine economy; and (3) to put forward practical optimization paths and safeguarding measures and provide a decision-making reference for the implementation of China’s marine development strategy. The research innovations of this paper are as follows: (1) bring into the discussion the rigid constraints of resources and negative environmental effects, build a measurement model of the *GTFP* of the marine economy, and use the directional distance function and *GML* index as a reference to conduct useful research on the measurement of the *GTFP* of China’s marine economy; and (2) unlike previous endogenous perspectives based on input–output, this paper starts with exogenous factors, systematically takes into account the linear and nonlinear relationship between various factors and the *GTFP* of the marine economy, and deeply explores the optimization path of the *GTFP* of China’s marine economy.

## 2. The Mechanisms of Influencing Factors on the *GTFP* of the Marine Economy

**Figure 1:**
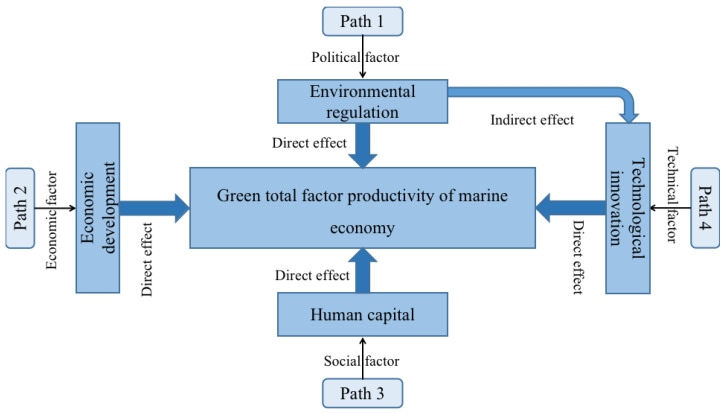
The impact path of influencing factors on *GTFP* of marine economy.

The *GTFP* of the marine economy is related to all aspects of the comprehensive system of “economy–environment–resources”. Economic development, structural factors, technical factors, energy factors, environmental regulations, and other factors are likely to have an impact on the *GTFP* of the marine economy. Considering the author’s research ability and the limitations of the measurement model, it is unrealistic to incorporate all the influencing factors into the research model. Therefore, after summarizing the existing literature and taking into account the availability of data, this paper systematically takes into account the direct and indirect effects; comprehensively considers the four paths of environmental regulations (political factor), economic development (economic factor), human capital (social factor), and technological innovations (technical factor); and qualitatively analyzes the internal mechanisms of the different factors that affect the *GTFP* of the marine economy (Figure 1).

### 2.1. The Direct Effect Mechanisms

(1) Environmental regulations

The consumption of resources will produce pollutants that will negatively impact economic development, social progress, and environmental protection. In order to realize the sustainable development of the “economy–society–environment” system, the government regulates the discharge of pollutants using emissions permits, administrative penalties, and the collection of emissions fees, which are known as environmental regulations [39].

Improvements in the intensity of environmental regulations will increase the costs of enterprise locations and pollution-treatment equipment, thus increasing the potential costs of starting enterprises, especially for pollution-intensive enterprises [8]. At the same time, they will increase the costs of environmental governance and the technical innovation of incumbent enterprises, speed up the elimination of pollution-intensive enterprises, and reduce the proportion of pollution-intensive enterprises, which promotes the upgrading of industrial structures [40]. In this way, factors such as capital and labor can be transferred from pollution-intensive industries with low productivity to industries using clean, environmentally protective technology and with high productivity so as to realize the reconfiguration of resources and improvements in environmental quality, which promotes the growth of the *GTFP* of the marine economy and the rapid development of high-tech environmentally protective industries [41]. These industries have a strong technology spillover effect and overall, they accelerate the upgrade to environmentally protective technologies and technical efficiency, thus promoting the *GTFP* of the marine economy [42].

In addition, the introduction of environmental regulation policies, especially market-motivated regulatory tools, such as environmental tax and energy tax, can effectively promote the marketization of the price of non-renewable resources, truly reflect the cost of environmental and other resources as well as the supply and demand situation, reduce the price distortions for non-renewable resources, and change the development model of the marine economy so as to promote the upgrading of factor structures, improve the efficiency of factor resource allocations, and improve the level and efficiency of green technology [43]. In short, the implementation of environmental regulation policies promotes the *GTFP* of the marine economy by forcing enterprises to change traditional modes of factor input and the structures of factor demand, which promotes the *GTFP* of the marine economy.

Based on the above analysis of the mechanisms through which environmental regulations influence the *GTFP* of the marine economy, this study proposes the following hypothesis:

**Hypothesis** **1** **(H1).** *Environmental regulations are positively related to the GTFP of the marine economy*.

(2) Economic development

On the one hand, there is no doubt that human beings develop natural resources through economic activities based on their own cultural and material needs [44]. With the development of the marine economy, the demand and consumption of marine resources in China have been showing an upward trend [45,46]. Due to technical reasons, the consumption of marine resources has been wasted and inefficient for a long time, which has seriously hindered the growth of the *GTFP* of the marine economy [18].

On the other hand, with the rapid development of the marine economy, a large number of environmental pollutants have been formed [6]. When the quantity or type of pollutants exceeds the self-purification capacity that the environment can accommodate, it will lead to a decline in environmental quality and could trigger a vicious cycle in the marine ecosystem, which has become an important part of restricting the growth of the *GTFP* of the marine economy [47].

Based on the above analysis of the mechanisms through which economic development influences the *GTFP* of the marine economy, this study proposes the following hypothesis:

**Hypothesis** **2** **(H2).** *Economic development is negatively related to the GTFP of the marine economy*.

(3) Human capital

The development of the marine economy cannot be separated from the support of human capital. The quality of human capital is a major factor in improving the *GTFP* of the marine economy.

First, scientific and technological progress is the core driving force for the transformation of the modes of marine economic development, which helps to improve the quality of marine economic development. Human capital can promote scientific and technological progress through research and development (R&D); create new market demand; and improve the investment, demand, and consumption structures, which then accelerates the optimization and upgrade of industrial structures, brings high-quality and effective supply and promotes the improvement of *GTFP* [48].

Second, technological capability can be regarded as the stock of technological knowledge that a country possesses to effectively participate in competition, whereas human capital is the endogenous source of the upgrade of the stock of technological knowledge and is also the key index for measuring the level of scientific research activities in a country [49]. Increasing human capital can significantly improve the technological knowledge stock of a country and promote the positive effects of knowledge spillover [50]. Human capital can provide new information resources and technological knowledge for technological innovation. After a period of R&D, scientific research input will be transformed into achievements in technological knowledge and the knowledge stock with independent R&D capacities will accumulate. At the same time, human capital can also effectively promote the ability to absorb spillover from the external environment, supplement and replace external and internal resources, form new knowledge, and continuously promote the renewal of knowledge stock so as to promote *GTFP* [51].

Third, the significance of technology diffusion is to popularize the achievements of technological innovation, improve the level of productivity, and promote the *GTFP* of the marine economy. Schumpeter believes that the essence of technology diffusion is the imitation of advanced technology, and human capital is one of the indispensable prerequisites for technological imitation [52]. From the standpoint of the human capital reserve, human capital stock is the key factor for absorbing technological capabilities. A shortage of human capital reserves will directly affect the occurrence and formation of technology diffusion. From the standpoint of the professional quality of human capital, highly skilled technicians with professional knowledge are closely integrated with technology diffusion resources as they interact with each other [53]. When the technological invention comes into being, it will not only attract other enterprises to follow and imitate but also force improvements and secondary innovations based on the original technology, which will lead to technological spillover [54]. Therefore, under the technology spillover effect, an increase in human capital can effectively improve the number and scale of technology-intensive enterprises, guide the reform of traditional enterprises, realize the diffusion and dissemination of new high-quality technologies, and thus promote the growth of the *GTFP* of the marine economy.

Based on the above analysis of the mechanisms through which human capital influences the *GTFP* of the marine economy, this study proposes the following hypothesis:

**Hypothesis** **3** **(H3).** *Human capital is positively related to the GTFP of the marine economy*.

(4) Technological innovation

Due to harsh and changeable natural marine conditions and insufficient understanding of the ocean, the marine economy requires more technological innovation than the land economy and its technology-intensive attributes are stronger.

On the one hand, due to the preferential use of technological innovations in a small number of enterprises, these enterprises have not only improved production efficiency but also reduced production costs, thus making their products more competitive in the market [55]. In this way, the production factors in the market will gradually transfer to the enterprises with high production efficiency, forcing other enterprises with low production efficiency to adopt advanced technologies to gain market competitiveness so as to promote the *GTFP* of the marine economy [8].

On the other hand, technological innovations can improve on the original production technology, change the input proportion of production factors, and replace relatively scarce production factors with relatively abundant production factors [8,56]. At the same time, with the application of new technologies, the original production skills mastered by laborers can no longer meet the needs of production, forcing laborers to re-learn production skills that are needed by new technologies so as to meet the needs of production [57]. To a certain extent, this improves the quality of laborers. In the production of equivalent products, the output of pollutants is reduced and the *GTFP* of the marine economy is improved.

Based on the above analysis of the mechanisms through which technological innovations influence the *GTFP* of the marine economy, this study proposes the following hypothesis:

**Hypothesis** **4** **(H4).** *Technological innovation is positively related to the GTFP of the marine economy*.

### 2.2. The Indirect Effect Mechanism

Environmental regulations have an indirect impact on the *GTFP* of the marine economy through technological innovations. This indirect impact is established on the premise that environmental regulations have an impact on technological innovations and technological innovations have an impact on the *GTFP* of the marine economy [58]. Since this paper has analyzed the mechanisms through which technological innovations influence the *GTFP* of the marine economy in the previous section, this section mainly analyzes the impact of the mechanisms of environmental regulations on technological innovations.

Through technological innovations, environmental regulations have an uncertain impact on the *GTFP* of the marine economy, with both negative “offset effects” and positive “compensation effects” [8]. On the one hand, in order to restrict the behavior of enterprises, the government has formulated a series of environmental regulation policies. In order to comply with the government’s environmental regulation standards, enterprises must take corresponding measures. These pollution control measures will increase the production costs of enterprises. In the event that total costs remain unchanged, the increased costs of pollution control due to the response to environmental regulation policies will inevitably form the majority of the technological innovation costs of enterprises, which forms the “offset effect” of environmental regulations, thus hindering the improvement of the *GTFP* of the marine economy [59]. On the other hand, once the government’s environmental regulation policy has become the norm, enterprises have to accept the requirements of the environmental regulations, incorporate technological innovations, and transform the past production model with high pollution and high emissions into an environmentally friendly production model so as to improve the sustainable development ability of enterprises. Environmental regulations force enterprises to incorporate technological innovations and improve processes, which improves the market competitiveness of enterprises, increases corporate profits, partially or fully offsets the costs of pollution control, and forms the “innovation compensation effect” of environmental regulations, which promotes the improvement of *GTFP* [60]. In conclusion, due to this indirect effect mechanism, the direction of the net impact of environmental regulations on the *GTFP* of the marine economy is uncertain as it depends on the sum of two effects and needs to be tested empirically [61].

Based on the above analysis of the indirect effect mechanism, this study proposes the following hypothesis:

**Hypothesis** **5** **(H5).** *When the intensity of environmental regulations crosses a certain threshold, the impact path of environmental regulations on the GTFP of the marine economy will change. In other words, there is a nonlinear relationship between environmental regulations and the GTFP of the marine economy*.

## 3. Methodology and Data

### 3.1. Methodology

(1) The measurement model of the *GTFP* of the marine economy

Oh proposed a global production possibility set $P^{G}=P^{1}\cup P^{2}\cup \cdots \cup P^{T}$ and constructed the Global Malmquist–Luenberger (*GML*) index, which can effectively avoid the defect of no solution to linear programming [62,63]. At the same time, this continuous production frontier avoids the possibility of the inward shifting of the production frontier, that is, it can avoid the possibility of the phenomenon of “technical retrogression”, thus avoiding the “passive” improvement of production efficiency. The *GML* index for the period *t* to *t* + 1 is defined as follows.

(1) $$\begin{array}{l} GML_{t}^{t+1}=\frac{1+{\vec{D}}_{o}^{G}(x^{t},y^{t},b^{t};y^{t},-b^{t})}{1+{\vec{D}}_{o}^{G}(x^{t+1},y^{t+1},b^{t+1};y^{t+1},-b^{t+1})}\\ =\frac{1+{\vec{D}}_{o}^{t}(x^{t},y^{t},b^{t};y^{t},-b^{t})}{1+{\vec{D}}_{o}^{t+1}(x^{t+1},y^{t+1},b^{t+1};y^{t+1},-b^{t+1})}\cdot \frac{\left(1+{\vec{D}}_{o}^{G}(x^{t},y^{t},b^{t};y^{t},-b^{t}))/(1+{\vec{D}}_{o}^{t}(x^{t},y^{t},b^{t};y^{t},-b^{t})\right)}{\left(1+{\vec{D}}_{o}^{G}(x^{t+1},y^{t+1},b^{t+1};y^{t+1},-b^{t+1}))/(1+{\vec{D}}_{o}^{t+1}(x^{t+1},y^{t+1},b^{t+1};y^{t+1},-b^{t+1})\right)}\\ =GMLEC_{t}^{t+1}\cdot GMLTC_{t}^{t+1} \end{array}$$

Among them, *D^t^* and *D^G^* represent the directional distance function based on the same period and the global production possibility set, respectively. *GML* represents the change in the green total factor productivity of decision-making units in two adjacent periods; *GML* > 1 represents the improvement of efficiency, and *GML* < 1 represents the decline of efficiency.

(2) The establishment of a direct effect model

Taking the *GTFP* of the marine economy as an explained variable and marine environmental regulations, marine economic development, marine human capital, and marine technological innovations as the explanatory variables, the following regression model is constructed to test the direct effect of the influencing factors on the *GTFP* of the marine economy.

(2) $$GTFP_{it}=\alpha +\beta_{1}ER_{it}+\beta_{2}{\mathrm{DEV}}_{it}+\beta_{3}HUMAN_{it}+\beta_{4}TECH_{it}+\mu_{i}+\varepsilon_{it}$$

Among them, $GTFP_{it}$ represents the *GTFP* of the marine economy, $ER_{it}$ represents marine environmental regulations, ${\mathrm{DEV}}_{it}$ represents marine economic development, $HUMAN_{it}$ represents marine human capital, $TECH_{it}$ represents marine technological innovations, $\mu_{i}$ is individual heterogeneity, which is used to control regional differences, and $\varepsilon_{it}$ is the disturbance term.

(3) The establishment of the threshold effect model

The existing literature mainly studies the nonlinear relationship in three ways. One is to introduce a dummy variable into the model, which is analyzed through the intersection term of the explanatory variable and dummy variable. The second is to introduce the quadratic or cubic terms of the explanatory variable into the model and investigate the mutation point between them through the higher terms of the explanatory variable. The third is group regression analysis, which is studied by regression analysis of explanatory and explained variables at different intervals. However, the introduction of the quadratic, cubic, or intersection terms of the explanatory variables in the first two methods may cause serious collinearity, thus affecting the scientificity and accuracy of the results. The group regression analysis method, namely the threshold model, can overcome the shortcomings of the above two models and scientifically analyze the relationship between the core variables at different intervals. Therefore, this paper uses Hansen’s threshold regression to construct a nonlinear structural model in order to obtain the optimal applicable range for the intensity of regulations [64]. The threshold model is essentially looking for one or more critical points and divides them into multiple intervals according to the critical points to observe the differences in the coefficients within the interval. The basic model is as follows.

Single threshold model:

(3) $$\begin{array}{ll} GTFP_{it}= & \delta_{0}+\alpha_{1}DEV_{it}+\alpha_{2}HUMAN_{it}+\alpha_{3}TECH_{it}+\beta_{1}ER_{it}•I(ER_{it}\leq \gamma_{1})\\ & \;+\beta_{2}ER_{it}•I(ER_{it}>\gamma_{1})+\varepsilon_{it} \end{array}$$

Multiple threshold model:

(4) $$\begin{array}{ll} GTFP_{it}= & \delta_{0}+\alpha_{1}DEV_{it}+\alpha_{2}HUMAN_{it}+\alpha_{3}TECH_{it}+\beta_{1}ER_{it}•I(ER_{it}\leq \gamma_{1})\\ & +\beta_{2}ER_{it}•I(\gamma_{1}<ER_{it}\leq \gamma_{2})+...+\beta_{n}ER_{it}•I(ER_{it}>\gamma_{n})+\varepsilon_{it} \end{array}$$

where γ is the threshold value to be calculated, I(•) is the indicative function, and $\varepsilon_{it}$ is the disturbance term.

### 3.2. Variable Selection and Data Interpretation

The index data adopted in this paper include 11 coastal provinces and cities in China, covering the period from 2006 to 2018. The data are from the “China Statistical Yearbook”, “China Marine Statistical Yearbook”, the website of the National Bureau of Statistics, and the author’s own measurements. In order to maintain the comparability of the data, the variables involving price factors in this study have been deflated with 2006 as the base period. Table 1 presents the basic characteristics of the indicators.

(1) *GTFP* of the marine economy

This paper selects marine capital stock, national ocean-related employed personnel by region, quay length, number of travel agencies in coastal areas, confirmed sea area, and energy consumption as the input indicators [2,65,66,67,68]. It is worth mentioning that the energy consumption of the marine economy in this paper is calculated by multiplying the total energy consumption of each coastal region by the proportion of the GOP in the Gross Regional Product (GRP) [2]. As for the desirable output indicator, this paper selects GOP [18]. As for the undesirable output indicators, this paper selects the emissions of marine wastewater, marine waste gas, and marine solid wastes and integrates the emissions of marine industries’ “three wastes” into a comprehensive index of environmental pollution through the entropy method [8,69,70,71]. The larger the indicator, the more serious the marine environment pollution. Finally, this paper uses the *GML* index and MATLAB software to measure the *GTFP* of the marine economy, which is expressed by *GTFP*.

(2) Marine environmental regulations

Based on the two types of environmental regulation tools in formal regulations, this paper chooses the corresponding representative indicators to comprehensively reflect the intensity of marine environmental regulations. Among them, the unit output value of investment in marine pollution control completion is taken as the indicator to measure the “command-regulation-type” regulatory tool, which represents the costs to governments or enterprises to ensure that pollutant emissions meet the standards. The larger the numerical value, the higher the cost of pollution reduction and the stronger the environmental regulation intensity. Sea area royalties levied by unit area are taken as the indicator to measure the “economic-motivation-type” regulatory tool. The larger the value, the stronger the environmental regulation.

Unit output value of investment in marine pollution control completion = Investment in industrial pollution control completion × The proportion of the marine industry/Gross ocean product(5)

Sea area royalties levied by unit area = Levy of sea area royalties/Confirmed sea area by the government(6)

(3) Marine economic development

Based on the processing method proposed by Bin et al. [72], this paper uses the proportion of GOP to GDP in coastal regions as the representative indicator of marine economic development, which is expressed by *DEV*.

(4) Marine human capital

Based on the processing method of Romer [73], this paper selects the proportion of professional and technical personnel in marine scientific research institutions to all ocean-related employed personnel to reflect the quality of marine human capital, which is expressed by *HUMAN*.

(5) Marine technological innovation

This paper chooses the total number of invention patents owned by regional marine scientific research institutions (units: pieces) as the proxy variable to measure marine technological innovation, which is expressed by *TECH* in the formula.

## 4. Results

### 4.1. Analysis of the GTFP of China’s Marine Economy

**Figure 2:**
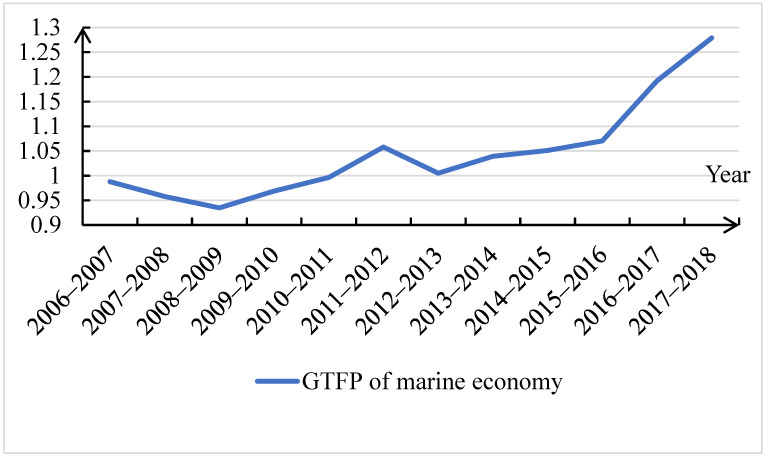
Dynamic trends of *GTFP* of China’s marine economy from 2006 to 2018.

This paper uses the *GML* index and MATLAB software to measure the *GTFP* of China’s marine economy from 2006 to 2018. As shown in Figure 2, during the sample period, the overall *GTFP* of China‘s marine economy was in a state of improvement and the *GML* index increased from 0.9878 in 2006 to 1.2789 in 2018. This is closely related to the Chinese government’s emphasis on the marine economy as well as a series of measures such as the adjustment of marine industrial structures and the proposal of a maritime power strategy. Specifically, 2006 was in the early stages of the “Eleventh Five-Year Plan”. Facing pressures on marine resources and the environment, China began to change the mode of economic development and strengthen the protection of resources and the environment. The development of the marine economy began to focus on quality and efficiency. However, due to the fragility of the marine ecological environment and the serious discharge of wastewater and residues caused by accumulation in the early stages, the positive effects on the marine economy could not be manifested immediately, resulting in a slight decrease in the *GTFP* of the marine economy. In 2008, affected by the global financial crisis, developments in imports, trade, investments, and the marine industry were hindered, resulting in a cliff-like decline in the *GTFP* of the marine economy, which showed significant negative growth. After 2009, due to strong macro-control by the government, the *GTFP* of the marine economy returned to a positive growth state and this trend continued until 2012. This is due to four reasons: First, since 2008, China has actively promoted new mechanisms for the comprehensive management of marine science and technology. For example, the marine science and technology departments of 11 coastal provinces and cities established a “joint work system”, which promoted the complementary and coordinated development of marine science and technology and industry among the coastal areas. Second, during the “Eleventh Five-Year Plan” period, the state greatly increased investment in marine science and technology and successively launched the “National Science and Technology Support Plan”, the “863 Plan”, and the “973 Plan” in the marine field. Third, in 2009, the state promulgated the “Island Protection Law of the People’s Republic of China”, which proposed “protecting the ecosystem of islands and their surrounding waters, rationally developing and utilizing island natural resources, safeguarding national marine rights and interests, and promoting sustainable economic and social development”. Fourth, \ in the “Twelfth Five-Year Plan” in 2011, the Chinese government stated that “we should adhere to the overall planning of land and sea, formulate and implement marine development strategies, and improve the ability of marine development, control and comprehensive management”. These measures helped to improve the *GTFP* of the marine economy in coastal areas. Although China attaches great importance to the protection of the marine ecological environment, extensive traditional marine economic production activities for pursuing marine economic output that follow a “pollution first, treatment afterwards” way of thinking still exist. At the same time, the “Bulletin on the State of China’s Marine Ecological Environment (2013)” showed that “the pressure of land-based sewage discharge was still huge, some coastal areas were seriously polluted, and the problems of marine habitat degradation and frequent environmental disasters were still prominent”. The extensive development path of the marine economy coexisted with the pressures of marine ecological environment protection, which was an important reason for the overall fluctuation and downward trend of the *GTFP* of the marine economy from 2012 to 2013. Afterward, with the proposal and promotion of the maritime power strategy, the *GTFP* of the marine economy began to gradually rise. In addition, the accumulated results of previous investment in scientific research gradually emerged. Progress in marine science and technology promoted the transformation and upgrade of industrial structures in coastal areas. By 2015, most areas had realized the advanced industrial structural model of “Three Two One” and the development of the marine economy became more efficient.

**Figure 3:**
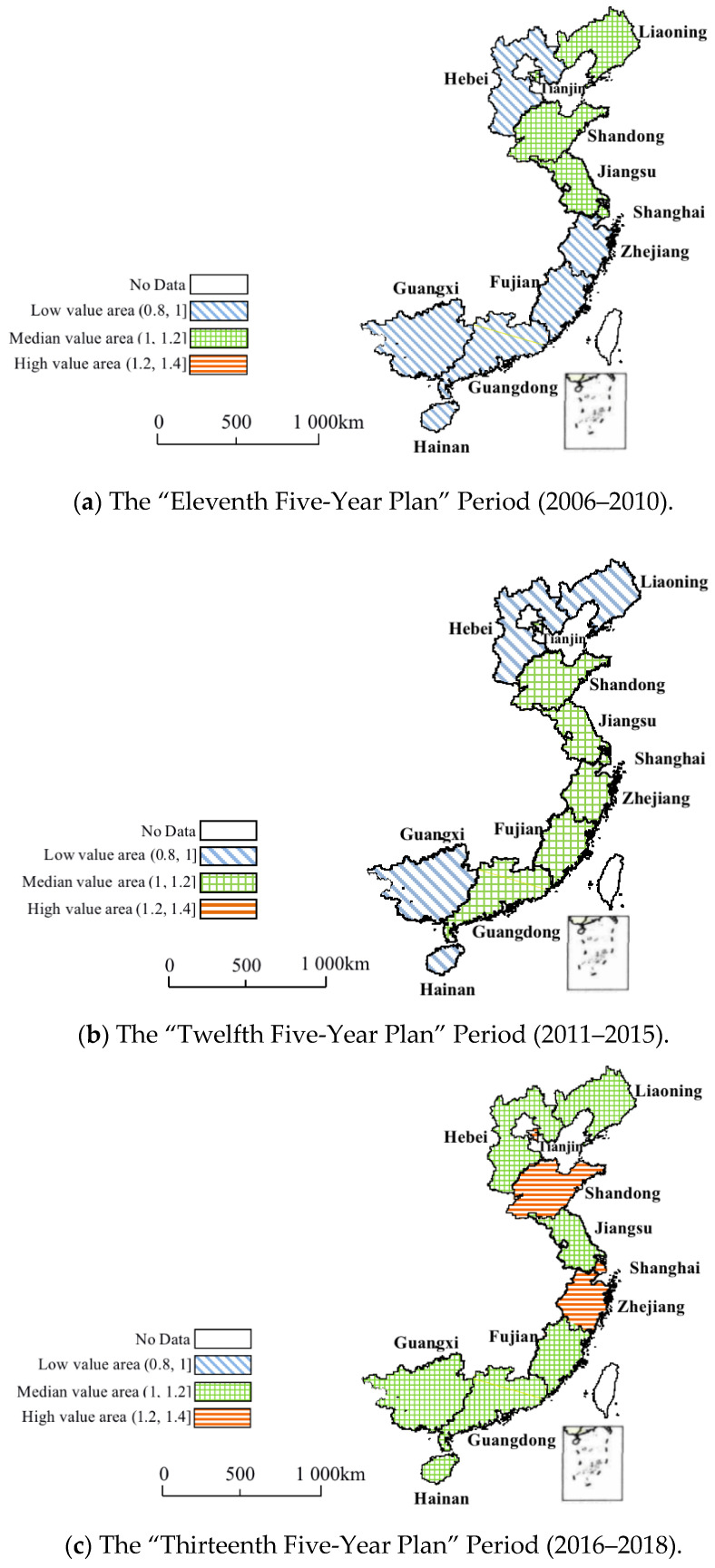
Regional changes of the *GTFP* of China’s marine economy from 2006 to 2018.

This paper further divides the *GTFP* of the marine economy of 11 coastal provinces and cities into three intervals, namely low-value areas, medium-value areas, and high-value areas. The comparative analysis of the coastal areas is carried out in three time periods: the “Eleventh Five-Year Plan” Period (2006–2010), the “Twelfth Five-Year Plan” Period (2011–2015), and the “Thirteenth Five-Year Plan” Period (2016–2018). As shown in Figure 3, during the “Eleventh Five-Year Plan” period (2006–2010), the *GTFP* of China’s marine economy showed a pattern of “strong in the north, weak in the south and rising in the middle”, among which Liaoning, Tianjin, and Shandong maintained a good momentum of development, and Shanghai and Jiangsu rose as the new forces. With the increasing national attention on the ocean, China’s marine economy ushered in a new round of development opportunities. During the “Twelfth Five-Year Plan” period (2011–2015), in addition to Tianjin, Shandong, Shanghai, and Jiangsu remaining in the medium-value areas, the *GTFP* of the marine economy in Zhejiang, Fujian, and Guangdong also showed an obvious upward trend. This could be due to the State Council’s approval of pilot projects for marine economic development in these provinces and cities. Focusing on regional characteristics and actual needs, the pilot areas made bold innovations and positive practices, explored the establishment of a leadership system and working mechanisms, and formulated a series of supporting policies and financial measures. In addition, during this period, the *GTFP* of the marine economy in Liaoning fell in the low-value areas, which could be due to the large proportion of traditional marine industries and the small proportion of emerging marine industries. Only the proportion of the output value for marine fishery and marine shipping industries was greater than 50%. The proportion of marine biological medicine and the comprehensive utilization of seawater, marine power generation, and other industries to GOP was less than 1% [9]. In 2012, there were 17 marine scientific research institutions, ranking fourth place; 865 R&D workers, ranking sixth place; CNY 3.96628 million of internal expenditure on R&D, ranking sixth place; and 205 R&D projects, ranking eighth place. Obviously, low inputs in marine science and technology not only worked against the improvement of the overall level and efficiency of comprehensive marine economic development but also affected the enthusiasm for technological innovations in marine enterprises and the transformation rate of marine scientific and technological achievements. During the “Thirteenth Five-Year Plan” Period (2016–2018), relying on improvements in technological progress and technical efficiency, the marine economy of 11 coastal provinces and cities showed a good development trend and regional differences gradually narrowed. This not only resulted from the adjustment of marine industrial structures but also from the country’s strategic commitment to adhering to the overall planning of land and sea environments and developing maritime power. It is worth mentioning that the *GTFP* of the marine economies in Tianjin, Shandong, Shanghai, and Zhejiang have risen to that of a high-value area. The construction of Tianjin Binhai New Area and the Zhejiang Zhoushan Archipelago New Area has promoted the development of the marine economy of these two areas to a certain extent. Through the active development of marine engineering construction and marine biomedical emerging industries, the *GTFP* of the marine economy in these two areas has increased rapidly. In addition, the Beijing–Tianjin–Hebei integration process has also accelerated the investment and utilization rate of marine science and technology in Tianjin, and Tianjin’s marine economic development has achieved remarkable results. Shandong is a relatively developed area of marine science and technology in China, especially with nearly 50% of the country’s marine talents. The establishment of the Shandong Peninsula Blue Economic Zone has laid a solid foundation for the green development of its marine economy. In addition, coastal cities in Shandong pay attention to relevant opportunities and policies, strive to improve the scientificity and rationality of the development and utilization of marine resources, and constantly promote the rapid development of high-tech marine industries, resulting in remarkable results in the green development of the marine economy. Shanghai has a solid economic foundation and an advantageous geographical location. The shipping and transportation industries are far advanced. Coupled with the continuous development of emerging industries such as marine renewable energy utilization and marine biomedicine, Shanghai has gradually formed a unique marine industry system. At the same time, since the 21st century, Shanghai has continuously promoted the three-year action plan for environmental protection, effectively improved the pollution of the marine ecological environment, and achieved remarkable results in green development.

### 4.2. Empirical Test of the Impact Path on the GTFP of China’s Marine Economy

In order to eliminate the interference of factors such as heteroscedasticity, this study adopts logarithmic processing for all data.

(1) Stationarity test

For non-stationary data, a common change in trends sometimes appears and although regression analysis could show better results, it has no practical significance. Therefore, this study first performs a stationarity test on the panel data. The traditional way to judge whether the data is stationary is to test the unit root of the data. There are four specific unit root test methods: the LLC test, IPS test, Fisher-ADF test, and PP-Fisher test. The original hypothesis of these tests is that there is a unit root. If the conclusion rejects the original hypothesis, it indicates that the data is stationary. The specific results obtained through the tests are shown in Table 2 below. It can be seen from the tests that all variables have passed the stationarity test after the first-order difference. Subsequently, in order to avoid the phenomenon of spurious regression, this paper uses the Kao test to conduct a cointegration test. The results show that the original hypothesis is rejected at the 5% significance level, that is, there is a long-term and stable equilibrium relationship between the panel data.

(2) Model estimation and test

Before the regression analysis, this paper uses the variance inflation factor (VIF) to test the multicollinearity of each explanatory variable. The results show that the VIF value of each variable and the average VIF value of the explanatory variable are far less than 10, so there is no multicollinearity among the variables. In addition, considering that the panel data may have heteroscedasticity and autocorrelation, this paper applies the modified Wald test, Wooldridge test, and Pesaran’s test to the model and the results show that all tests reject the original hypothesis at the 1% significance level (Table 3).

In order to reach a more robust conclusion, this paper discriminates the best estimation method through the following test: the LM test is used to compare the optimal model of mixed OLS regression and random effect models, the F test is used to compare the optimal model of mixed OLS regression and fixed-effect models, and the Hausman test is used to compare the optimal model of fixed effect and random effect models. The specific results are as follows (Table 4).

Therefore, it is appropriate to select a random effect for the model estimation, and feasible generalized least-square estimation (FGLS) is also a random effect estimator, which has the same positive and negative correlation with the results of the random effect estimation and is significantly better than the random effect. In addition, this method has advantages in eliminating the effects of intra-group autocorrelation and inter-group heteroscedasticity on the results of the model. In summary, this paper considers that the FGLS estimation has a certain degree of robustness.

(3) Direct effect of influencing factors on the *GTFP* of the marine economy

Based on the above model estimation, this paper can quantitatively analyze the direct effect of the influencing factors on the *GTFP* of the marine economy and specific conclusions can be drawn as follows (Table 5).

The regression coefficient of environmental regulations on the *GTFP* of the marine economy is negative and it passes the significance test of 1%, indicating that Hypothesis 1 is not established. Compared with developed countries, China’s marine economy is still in its infancy and its technological level is relatively low, resulting in its strong dependence on the marine environment and resources. This means that strict environmental regulations may impose more constraints on the development of the marine industry. In order to meet the requirements of environmental protection regulations, marine-related enterprises have to use factor resources for environmental governance, which squeezes productive funds, increases costs, reduces enterprise production efficiency and industrial efficiency, and ultimately has a negative impact on the *GTFP* of the marine economy.

The regression coefficient of economic development is 0.4129, which indicates that for every 1 percentage point increase in economic development, the *GTFP* of the marine economy will increase by 0.4129 percentage points. On the one hand, with developments in the marine economy and improvements in people’s living standards, people’s awareness of environmental protection is gradually enhanced and the requirements for environmental quality are increasingly improved, which further optimizes the industrial structure, thus promoting the *GTFP* of the marine economy. On the other hand, it also shows that improvements in economic development provide the rich material basis for scientific and technological innovation, thus promoting technological progress, which ultimately plays a positive role in promoting the *GTFP* of the marine economy. Hypothesis 2 is not established.

The regression coefficient of human capital is positive and passes the significance test but the coefficient is very small. It indicates that the number of professionals engaged in marine research and development accounts for a small proportion of ocean-related employed personnel. There are still some practical problems with marine professionals, such as supply–demand asymmetry, the relatively small cultivation scale, and poor matching between the professional setting and actual need, which shows that increasing the cultivation scale of marine professionals and exploring reasonable modes of personnel cultivation are important tasks for improving the *GTFP* of China’s marine economy. Hypothesis 3 is established.

The regression coefficient of technological innovation is positive and passes the significance test but the coefficient is very small, which shows that technological innovation does not play a significant role in the growth of the *GTFP* of China’s marine economy. Based on current national conditions, the theory that technological innovation has a positive role in promoting TFP has been tested in the practice of economic development. However, in the process of the development of the marine economy, technology innovation has not gained an absolute advantage in competition with the substitution of factor input, which may be affected by the following two reasons. The first is the application benefits of technological innovations. At present, China’s marine economy still has the problem of low benefits from technology innovation and application; the achievements in technological innovation are not suitable for the domestic market and are difficult to use. Compared with factor input, the economic benefits lack an absolute advantage. The other is the process characteristics of technological innovations. There is a time lag between the interaction with technological innovations and the growth of the marine economy. Technological innovation is a multi-stage and multi-factor value chain transmission process from the input to the output of technological innovations and then to the realization of the achievements of technological innovations. This not only means that the benefits of technological innovations cannot be reflected at present but also means that some technological innovation projects with significant long-term benefits have to be selectively shelved in favor of the company’s short-term return projects. Hypothesis 4 is established.

(4) Threshold effect of environmental regulations on the *GTFP* of the marine economy

The above discusses the impact of different influencing factors on the *GTFP* of the marine economy from a linear perspective. The following constructs a panel threshold model with environmental regulations as the threshold variable and attempts to explore the nonlinear threshold characteristics of the impact of environmental regulations on the *GTFP* of the marine economy. Before performing the threshold model regression, the specific form of the panel threshold model should be determined. The test results of 500 self-sampling times are shown in Table 6. It can be seen from the F statistic and the *p*-value that the triple threshold of environmental regulations fails the significance test, whereas the single and double thresholds pass the significance level test. Therefore, this paper adopts the double threshold model to analyze the threshold characteristics of the impact of environmental regulations on the *GTFP* of the marine economy.

After passing the significance test of the threshold effect, it is necessary to identify the authenticity of the threshold value. Table 7 shows the estimated threshold value and 95% confidence interval. It can be seen that the double thresholds for environmental regulations to promote the growth of the *GTFP* of the marine economy are 3.07 and 4.23 and the 95% confidence interval is [3.03, 3.45] and [4.18, 4.52].

Table 8 shows the threshold effect of environmental regulations on the *GTFP* of China’s marine economy during the sample period. The results show that environmental regulations in the three intervals have an impact on the *GTFP* of the marine economy, showing a “significant positive-significant negative-significant positive” trend, which verifies that there is an “inclined N-type” double threshold relationship between environmental regulations and the *GTFP* of the marine economy. Hypothesis 5 is established. At the same time, it also shows that the impact of environmental regulations on the *GTFP* of the marine economy depends on the intensity of environmental regulations and different intensities of environmental regulations have different dominant levels of the “innovation compensation effect” and “offset effect” that affect the *GTFP* of the marine economy. The establishment of the “Porter Hypothesis” is based on the premise that the intensity of environmental regulations is appropriate and that going beyond the limit is as bad as falling short. Therefore, it is necessary to gradually realize a benign balance between environmental regulations and economic development, comprehensively examine the bearing capacity of enterprises, avoid falling into a low-level equilibrium in the area of environmental regulations, and strive to minimize the negative impact.

(5) Robustness test

The robustness test is generally carried out by re-selecting the estimation method and replacing some key variables. If the re-estimated results do not differ significantly, it proves the robustness of the research results. In view of the problems of panel data such as inter-group heteroscedasticity, intra-group autocorrelation, and inter-group synchronous correlation, this paper uses FGLS to estimate the model when testing the direct effect of each influencing factor on the *GTFP* of the marine economy. Driscoll and Kraay (1998) proposed a progressively effective nonparametric covariance matrix estimation method in the case of N→∞ to solve the problems of heteroscedasticity and autocorrelation [74]. In view of this, this paper tests the robustness of the direct impact model by changing the regression method. The regression results are shown in Table 9. The results show that the sign and significance of the estimated coefficients of each variable are basically unchanged, indicating that the direct effect results are relatively robust.

This paper further tests the robustness of the threshold effect model by replacing the core explanatory variables in the model according to conventional practice to verify the robustness of the empirical study. Considering that China had already implemented the sewage charge system, it has a certain practical and theoretical basis. As a typical environmental regulation tool, the sewage charge is not only applicable to the general industry but also the marine industry. Therefore, this paper selects “the actual collection of sewage charges in various regions” as the substitute variable for environmental regulations and then reflects the environmental regulations of marine undertakings. The specific regression results are shown in Table 9. The relationship between environmental regulations and the *GTFP* of the marine economy is also an “inclined N-type” relationship and the signs for the other control variables remain unchanged, which proves that the threshold effect results in this paper are robust.

(6) Endogeneity test

Solving the endogenous problem is an aspect that cannot be ignored in empirical research. The causes of endogenous problems may include a bidirectional causal relationship between the explanatory variables and explained variables, the measurement error of indicators, or the omission of explanatory variables. The above empirical results verify the direction and intensity of environmental regulations, economic development, human capital, and technological innovations on the *GTFP* of the marine economy. However, the improvement of the *GTFP* of the marine economy may also affect environmental regulations, economic development, human capital, and technological innovations. Therefore, the bidirectional causal relationship between various factors and the *GTFP* of the marine economy may cause endogenous problems. In order to mitigate its impact on the research conclusion, this paper introduces the lag period of environmental regulations, economic development, human capital, and technological innovations as the explanatory variables, and uses the System Gaussian Mixed Model (system GMM) to test the model. The results are shown in Table 10. The results show that the regression coefficient and significance of the explanatory variables lagging one period are consistent with the basic model (2).

## 5. Discussion

The empirical results of this study have certain practical significance for the improvement path and policy formulation of the *GTFP* of China’s marine economy.

(1) Through empirical research, this study concludes that environmental regulations have an “inclined N” double-threshold effect on *GTFP*. The impact of environmental regulations on the *GTFP* of the marine economy depends on the intensity of environmental regulations, and different intensities of environmental regulation have different dominant levels of the “innovation compensation effect” and “offset effect” that affect the *GTFP* of the marine economy. Therefore, it is necessary to control the intensity of environmental regulations to a reasonable degree. Only in this way can the “innovation compensation effect” of environmental regulations be fully brought into play, thereby promoting the improvement of the *GTFP* of the marine economy. Based on this, coastal areas should fully consider the implementation conditions of the policy, refine the environmental regulation standards, and formulate a suitable environmental regulation policy system. Coastal areas with relatively serious environmental pollution should focus on “command-and-control” environmental regulations to reduce the intensity of pollution emissions, whereas coastal areas with relatively low pollution levels should flexibly apply “market-incentive” environmental regulations, such as environmental taxes, to improve their pollution control capabilities. In areas where marine economic development is relatively backward, economic development should be the top priority and since the elastic coefficient of environmental regulations is relatively small, it is necessary to adopt steadily strengthened environmental regulation policies. With the improvement of environmental awareness, some pollution-intensive industries in the more developed areas of the marine economy are gradually being transferred to other areas and the proportion of green industries is increasing. The government can consider prudently loosening the intensity of “market-incentive” environmental regulations and avoid implementing severe regulations that exceed their carrying capacity so as to reduce the negative impact on technological innovations. At the same time, the state should strengthen government and public supervision to prevent opportunism. In addition, the government also needs to coordinate local environmental regulation policies to avoid “pollution transfer” among regions due to inconsistent environmental standards and policy implementation.

(2) The above empirical results show that the positive impact of human capital on the *GTFP* of the marine economy has yet to be fully achieved. In this regard, the government should aim to improve training for marine experts to meet national and local strategic needs for marine expertise and accelerate the development of a diversified marine expertise training system dominated by marine-related scientific and educational institutions and vocational and technical colleges, supplemented by industry–university–research cooperation, domestic and foreign exchange, and cooperation and continuing education, to provide a strong foundation for improving the *GTFP* of the marine economy. In addition, focusing on the strategic goal of marine economic construction, the government should establish a group of strategic scientists who can break through key technologies, develop high-tech industries, drive emerging disciplines, and cultivate an innovative team of experts that can track developments in international marine technology and participate in international cooperation. On this basis, the government should strengthen the flexible introduction of experts. Identified high-level marine experts who are in short supply can be involved in a variety of ways, such as through part-time jobs, consultation, lectures, academic exchanges, technical contracting, technical cooperation and shareholding, investment in enterprises, and cooperative research so as to broaden the channels of talent recruitment, form a diversified pattern of ushering in new talent, and build a pool of marine experts.

(3) The theory that technological innovation has a positive role in promoting *GTFP* has been tested in economic development practices. However, in the field of marine economy, technological innovation has not yet achieved an absolute advantage. To this end, the government should improve the condition of the marine science and technology field, strengthen the research capabilities of the marine science and technology field, cultivate the independent innovation capabilities of the marine science and technology field, and make use of the combination of industry–university–research to continuously promote the transformation of scientific and technological achievements realize the potential efficiency of the relationship between technology innovations and the *GTFP* of the marine economy. Specifically, the first step would be to speed up the construction of marine scientific research bases, actively promote the construction of marine scientific data public service platforms, and implement comprehensive and multi-level marine information resource sharing and services. On this basis, the government should promote the research and formulation of marine technical standards with independent intellectual property rights and further improve the marine industry standards system. The second step would be to strengthen improvements to marine technology to realize the full impact of technology upgrades on *GTFP*. At the same time, the government should continue to strengthen the application capacity and allocation efficiency of existing technologies, promote cross-regional exchange and cooperation in the transformation and application of technologies, and achieve a growth state where technical efficiency is positively promoting *GTFP*. The third step would be to take marine-related enterprises as the main body, focus on strategic marine emerging industries, rely on market mechanisms, allow a guiding role of the government, build a number of strategic alliances for technological innovations in the marine industry, and cooperate in the R&D of key common technologies in the marine industry to enhance the core competitiveness of the regional marine economy.

## 6. Conclusions

For a long time, China’s marine economy has been relying on the input of capital, labor, and other factors to achieve wealth creation and economic growth through the expansion of scale. However, this mode of growth is ultimately insufficient and unsustainable. With the rise in labor costs and the strengthening of resource and environmental constraints, both the macro-economy and micro-individuals urgently need to find a new driving force for development to solve the problem of the insufficient momentum of growth in the traditional economy. The high-quality development of the marine economy emphasizes the dynamic balance in the “economy–society–ecological environment” system. To achieve this, continuously improving the *GTFP* of the marine economy has undoubtedly become the key point.

Therefore, this paper considers the rigid constraints of resources and negative environmental effects to construct a multi-factor evaluation model of the *GTFP* of the marine economy including capital, labor, and resources so as to expand the evaluation method system for the sustainable development of the marine economy. On this basis, this paper determines the influencing factors of the *GTFP* of China’s marine economy, qualitatively analyzes the mechanisms of each influencing factor on the *GTFP* of the marine economy, uses multi-dimensional data of coastal areas, and quantitatively analyzes the direct and indirect effects of the influencing factors on *GTFP*. Combined with the empirical results, this paper further discusses whether there is a “Porter Hypothesis” in the marine field and tries to unravel the mystery of the changes in the *GTFP* of the marine economy. The results showed that the *GTFP* of China’s marine economy was in a state of improvement, which increased from 0.9878 in 2006 to 1.2789 in 2018. The direct effects of environmental regulations on *GTFP* have a negative and significant impact, whereas economic development, human capital, and technological innovations have a positive and significant impact on *GTFP*. In addition, the nonlinear effect of environmental regulations on the *GTFP* of the marine economy shows a trend of “significant positive-significant negative-significant positive”, which verifies that there is an “inclined N-type” double-threshold relationship between environmental regulations and the *GTFP* of the marine economy. The impact of environmental regulations on the *GTFP* of the marine economy depends on the intensity of environmental regulations, and different intensities of environmental regulations have different dominant levels of the “innovation compensation effect” and “offset effect” that affect the *GTFP* of the marine economy. It is necessary to realize a benign balance between environmental regulations and economic development. By comprehensively examining the bearing capacity of enterprises and avoiding falling into a low-level equilibrium in the area of environmental regulations, it is possible to minimize the negative impact. Finally, this paper proposes an improvement path and countermeasures for the *GTFP* of the marine economy.

Due to objective factors such as data availability and research methods, this study also has some deficiencies. The research objects selected in this paper are 11 provinces and cities along the coast of China, which have a large spatial scale, and the research objects have not been deeply studied at the small-scale level. Future research could attempt to use 53 cities in the eastern coastal area as the research objects, more carefully investigate the *GTFP* of the marine economies in different cities, and further improve the research depth and practical significance of the existing study, which is also our next research direction.

## Figures and Tables

**Table 1:** The descriptive statistics analysis.

Variable Name	Variable Code	Mean	Standard Deviation	Minimum	Median	Maximum
*GTFP* of marine economy	*GTFP*	0.9981	0.1048	0.7691	0.9998	1.3589
Marine environmental regulations	*ER*	31.4513	19.24	6.1396	35.57	121.8921
Marine economic development	*DEV*	0.1792	0.0885	0.0524	0.1727	0.3459
Marine human capital	*HUMAN*	0.0004	0.0006	0.0001	0.0005	0.0016
Marine technological innovations	*TECH*	442.04	787.06	0	95	3771

**Table 2:** The unit root test.

Variable	Testing Method			
	LLC Test	IPS Test	Fisher-ADF Test	PP-Fisher Test
ΔLnGTFP	–12.7995 ***	–8.1829 ***	76.6564 ***	254.0961 ***
ΔLnER	–10.1735 ***	–4.0071 ***	104.7199 ***	42.0558 **
ΔLnDEV	–10.0837 ***	–3.9217 ***	58.0424 **	111.1468 ***
ΔLnHUMAN	–5.9792 ***	–2.0230 **	87.8138 ***	38.3127 ***
ΔLnTECH	–12.2443 ***	–4.3290 ***	88.2581 ***	110.9786 ***

Note: (1) “***”and “**” represent significance at confidence levels of 1% and 5% respectively. (2) Δ represents the first-order difference value of the variable.

**Table 3:** Heteroscedasticity and autocorrelation test.

Test	Test Object	Test Value	*p*-Value	Original Hypothesis	Whether to Accept the Original Hypothesis
Modified Wald test	Inter-group heteroscedasticity	791.64	0.0000	There is no inter-group heteroscedasticity	No
Wooldridge test	Intra-group autocorrelation	24.57	0.0000	There is no intra-group autocorrelation	No
Pesaran’s test	Inter-group synchronous correlation	7.79	0.0001	There is no inter-group synchronous correlation	No

**Table 4:** Test method discrimination.

Test Method	LM Test	F Test	Hausman Test
*p*-value	0.0000	0.0000	0.5981

**Table 5:** The direct effect of influencing factors on the *GTFP* of China’s marine economy.

Variable	Direct Effect
LnER	−0.0551 *** (−5.28)
LnDEV	0.4129 *** (3.36)
LnHUMAN	0.1602 *** (3.20)
LnTECH	0.0341 *** (6.62)

Note: (1) “***” represents significance at confidence levels of 1%. (2) Standard error is in brackets.

**Table 6:** Threshold effect test.

Model	F Value	*p* Value	BS Frequency
Single threshold	18.48 ***	0.001	500
Double threshold	9.36 ***	0.006	500
Triple threshold	2.51	0.323	500

Note: “***” represents significance at confidence levels of 1%.

**Table 7:** Estimated threshold value.

	Estimated Value	95% Confidence Interval
Threshold value $\gamma_{1}$	3.07	[3.03, 3.45]
Threshold value $\gamma_{2}$	4.23	[4.18, 4.52]

**Table 8:** The threshold effect of environmental regulations on the *GTFP* of China’s marine economy.

Variable	Threshold Effect
LnDEV	0.6327 *** (4.41)
LnHUMAN	0.2498 *** (3.78)
LnTECH	0.1009 *** (5.32)
LnER1 (*ER* ≤ 3.07)	0.1983 *** (3.45)
LnER2 (3.07 < *ER* ≤ 4.23)	−0.0789 *** (−4.93)
LnER3 (*ER* > 4.23)	0.2413 *** (6.61)

Note: (1) “***” represents significance at confidence levels of 1%. (2) Standard error is in brackets.

**Table 9:** Robustness test.

Variable	Direct Effect	Variable	Threshold Effect
LnER	−0.0124 *** (−3.67)	LnDEV	0.5991 *** (4.19)
LnDEV	0.6671 *** (5.21)	LnHUMAN	0.2774 *** (4.05)
LnHUMAN	0.1944 *** (3.38)	LnTECH	0.0956 *** (5.94)
LnTECH	0.0208 *** (5.63)	LnER1 (*ER* ≤ 0.334)	0.2139 *** (3.88)
		LnER2 (0.334 < *ER* ≤ 0.458)	−0.0859 *** (−4.52)
		LnER3 (*ER* > 0.458)	0.2563 *** (6.12)

Note: (1) “***” represents significance at confidence levels of 1%. (2) Standard error is in brackets.

**Table 10:** Endogenous test.

Variable	FGLS	Variable	System GMM
LnER	−0.0551 *** (−5.28)	LnER_1	−0.1417 *** (−5.84)
LnDEV	0.4129 *** (3.36)	LnDEV_1	0.6662 *** (3.89)
LnHUMAN	0.1602 *** (3.20)	LnHUMAN_1	0.2984 *** (3.71)
LnTECH	0.0341 *** (6.62)	LnTECH_1	0.0891 *** (5.43)
		Wald test	131.64 ***

Note: (1) “***” represents significance at confidence levels of 1%. (2) Standard error is in brackets.

## Data Availability

The data are from the “China Statistical Yearbook”, “China Marine Statistical Yearbook”, the website of the National Bureau of Statistics, and the author’s own measurements.
